# Performance of a through-transmit aberration correction method for transcranial focused ultrasound: Simulation and experimental evaluation

**DOI:** 10.1121/10.0043031

**Published:** 2026-03-01

**Authors:** Cooper L. Donovan, Charles F. Caskey

**Affiliations:** 1 Vanderbilt University Institute of Imaging Science, Vanderbilt University Medical Center, Nashville, Tennessee 37232, USA

## Abstract

Transcranial focused ultrasound is a promising neuromodulation tool, but skull-induced aberrations to the ultrasound beam require subject-specific correction. To predict and compensate for aberration, the standard in transcranial focused ultrasound is to perform acoustic simulations based on x-ray computed tomography images of the head. However, computed tomography-based methods face shortcomings due to imaging resolution and uncertainty in acoustic parameter estimates. Relativistic through-transmit (RTT) is a recently proposed imaging-independent method that corrects skull-induced aberrations by comparing through-skull acoustic transmission to free-field signals from two opposing transducer arrays. Although RTT has shown promise, its robustness across subjects and conditions has not been fully characterized. In this work, RTT performance was evaluated in simulation as a function of several parameters in four unique non-human primate subjects using commercial linear phased array transducers operating at 1 and 2 MHz. Simulated results were validated experimentally at 2 MHz. In simulations, RTT restored pressure amplitudes to within 5% of the intended value for three skulls but achieved only 60% of the intended pressure in one, with focal shift ranging 0.4 to 5.5 mm. RTT performance depended on signal-to-noise ratio and physical uncertainties and was markedly impacted by steering. Modifications to RTT are proposed and characterized.

## I. INTRODUCTION

Transcranial focused ultrasound (tFUS) is an emerging noninvasive neuromodulation tool that has shown promise as a non-addictive treatment modality for post-stroke chronic pain and other neurological pathologies (Darmani et al., 2022). However, beam aberration induced by the skull remains an obstacle for effective therapies. When an incident acoustic wavefront encounters the skull, complex acoustic interactions, including reflection, refraction, absorption, and scattering, attenuate the intensity of the acoustic focus and distort its shape and location as compared to the focus achieved in a homogeneous medium, such as water. Aberration correction is therefore an important step in achieving both scientific and clinical goals. For example, in order to elucidate the relationship between acoustic pressure and neuromodulatory effects of focused ultrasound, accurate and reliable methods for delivering a desired pressure to a specific target are necessary. Likewise, as safe exposure thresholds are established to mitigate the risk of deleterious effects, such as cavitation and heating (see Aubry et al., 2025), the intracranial pressure must be known accurately.

Various methods to correct for acoustic aberrations exist. The simple “derating” scheme relies on the assumption that the focal pressure achieved when sonicating in a water tank (the “free-field” pressure) is attenuated by a constant factor when transmitting transcranially (for a thorough explanation, see e.g., Martin et al., 2024). Transducer driving amplitudes can be scaled by the reciprocal of this factor, which can for example be estimated by water-tank measurements through *ex vivo* skulls to restore a desired pressure intracranially. For multielement therapeutic transducers, which generally comprise tens to hundreds of elements, each element can be driven individually with a unique phase and amplitude. Independent element control allows for more sophisticated multilayer derating schemes (e.g., Attali et al., 2023), but derating alone cannot account for the significant variability in attenuation that has been shown between subjects and even between targets within an individual subject (Gimeno et al., 2019). Derating also does not correct for phase aberration, and so it primarily compensates for amplitude losses rather than correcting the focal location.

Other methods, attempting to more accurately restore the ideal focal pressure and location while reducing side lobes and focal smear, rely on parameters derived from imaging modalities other than ultrasound. Commonly, x-ray computed tomography (CT) or ultrashort echo time magnetic resonance imaging of the head is used to estimate local acoustic parameters of the skull. By ray tracing or time-reversal simulation-based methods, corrective phases and amplitudes can then be estimated (Wang et al., 2025). These methods are currently standard (Leung et al., 2021), but suffer from uncertainties caused by the finite resolution of imaging (which in a clinical scanner is generally too coarse to capture the microstructure of the skull), the imperfect correlation between imaging values and acoustic propagation parameters (Clinard et al., 2025a, 2025b; Leung et al., 2019), and the inability to account for hair and imperfections in transducer-to-patient coupling. Imaging is also costly and CT requires exposure to ionizing radiation.

Riis et al. (2024a, 2024b), building on the work of Vignon et al. (2006), proposed a correction method called relativistic through-transmit (RTT) that does not rely on parameters from other imaging modalities. Instead, RTT uses only information acquired by transmitting and receiving on two transducer arrays that oppose each other across the skull. The method poses an inverse problem to extract amplitude and phase corrections from this propagation information. RTT has demonstrated proof-of-concept effectiveness in restoring pressure in *ex vivo* human skulls, and two pilot studies examined the use of tFUS neuromodulation for treatment of chronic pain and depression using RTT in human subjects (Riis et al., 2024c, 2025). As a relatively novel method, however, RTT’s performance remains to be rigorously characterized.

We probed RTT’s performance in simulation and experiment with respect to parameters that affect performance in real therapies and experiments, such as signal noise, positioning uncertainties, subject skull geometry, and intracranial target. To make use of commercially available transducers that are small relative to typical tFUS therapeutic arrays, we modeled rhesus macaque monkey skulls rather than human skulls. This choice maintained the approximate ratio of aperture width to skull diameter as compared to the RTT setup described in Riis et al. (2024a, 2024b). From these results, we identify areas for improvement in RTT. Finally, we propose modifications to the method that show evidence of better efficacy in these areas.

## II. METHODS

### A. Description of the correction method

The goal of aberration correction in general is to find an amplitude correction vector **x** and a phase correction vector τ that can be applied to the set of *N* uniform-amplitude element-wise driving signals $S(t)=\left\{s_{1}(t),\ldots ,s_{N}(t)\right\}$ (which are phased appropriately to form a focus in free-field) such that

(1) $$s_{i}^{\text{corrected}}(t)=x_{i}\cdot s_{i}\left(t+\tau_{i}\right).$$

The RTT method was originally proposed in Riis et al. (2024a, 2024b) and is recapitulated below as it was implemented in this work.

The transducers, each comprising *n* elements, were first placed opposite each other in a water tank. A 10-cycle pulse, windowed by a Gaussian, was emitted from a single transmitting element *i*, and time signals were received on a subset of elements on the opposing transducer. This subset was defined as all receiving elements for which the angle *θ*, between the vector connecting the transmitting element *i* to the receiving element *j* and the vector from element *i* to the tFUS target, was less than or equal to the acceptance angle of 15°. This particular angle was chosen as the result of an optimization described later in this work and therefore departs from the 10° acceptance angle used in the original method (Riis et al., 2024a, 2024b) (see Sec. IIIB and Fig. 4 for the optimization). Each received signal was dubbed $s_{ij}^{F}(t)$, where *F* denotes the free-field (water tank) case. This procedure was repeated for all elements on both transducers, so that all elements transmitted a single 10-cycle pulse once. The skull was then placed between the transducers without changing the transducers’ positions, and the same procedure as before was repeated to yield the set comprising time signals $s_{ij}^{B}(t)$, where *B* denotes the aberrated case.

The aberrated and free-field signals were then compared. An insertion loss factor $\xi_{ij}$ was defined as follows:

(2) $$\xi_{ij}=ln\;\left(\frac{max\left(s_{ij}^{B}(t)\right)}{max\left(s_{ij}^{F}(t)\right)}\right).$$

The logarithm is necessary to convert the multiplicative nature of acoustic absorption to an additive factor which can be represented easily in matrix form.

A time delay $\tau_{ij}$ was then selected as the delay which maximized the cross correlation between the pairs of signals $s_{ij}^{B}(t)$ and $s_{ij}^{F}(t)$.

Next, the many delays $\tau_{ij}$ and insertion loss factors $\xi_{ij}$ were arranged in two $1\times 2n^{2}$ vectors, where n was the number of elements on each of the two transducers. The problem of solving for the desired 1×2n vectors $\xi =\left[\xi_{1}\ldots \xi_{i}\ldots \xi_{2n}\right]$ and $\tau =\left[\tau_{1}\ldots \tau_{i}\ldots \tau_{2n}\right]$ could then be expressed in the following way (shown using ξ for example, process identical for τ):

(3) $$\left[\underset{2n^{2}\times 2n}{A}\right]\;\left[\begin{array}{c} \xi_{1}\\ \vdots \\ \xi_{i}\\ \vdots \\ \xi_{2n} \end{array}\right]=\left[\begin{array}{c} \xi_{1,n+1}\\ \vdots \\ \xi_{n,2n}\\ \xi_{n+1,1}\\ \vdots \\ \xi_{2n,n} \end{array}\right],$$

where *A* is the binary connectivity matrix that defines which transmit-receive pairs *i,j* were included in the problem. This inverse problem was solved by a Moore-Penrose pseudoinverse of the *A* matrix. The singular values were Tikhonov regularized after truncation, choosing the Tikhonov parameter *λ* that maximized the generalized cross-validation.

The pseudoinverse of *A* was dubbed *A*^+^, and the desired vectors were finally estimated from the recorded data as follows:

(4) $$A^{+}\;\left[\begin{array}{c} \xi_{1,n+1}\\ \vdots \\ \xi_{n,2n}\\ \xi_{n+1,1}\\ \vdots \\ \xi_{2n,n} \end{array}\right]\approx \left[\begin{array}{c} \xi_{1}\\ \vdots \\ \xi_{i}\\ \vdots \\ \xi_{2n} \end{array}\right].$$

The correction vector **x** was then as follows:

(5) $$x=\frac{1}{exp(\xi )}.$$

The vector τ is computed, independently from the computation of **x**, by similar means. The method described by Riis *et al*. employs an iterative approach to calculate the phases τ. In this work, we instead compute τ by finding *τ_ij_* analogously to Eq. (2) by choosing the offset that maximizes the normalized cross correlation between $s_{ij}^{B}(t)$ and $s_{ij}^{F}(t)$ in a single transmit-receive sequence. We then invert the $2n^{2}\times 1$ vector of *τ_ij_* values in the same manner as Eq. (4) to yield the 2*n* delays τ. This was done after early testing revealed instability in the iterative method and reduced the total computation time required for simulations by approximately a factor of ten. The supplementary material shows a representative case of high single-iteration correlation coupled with long-term instability.

### B. Simulation methods

First-order 2D acoustic simulations of RTT were performed using the k-Wave toolbox (version 1.4) in matlab R2019b (The MathWorks, Inc., Natick, Massachusetts) (Treeby and Cox, 2010). The provided CUDA binaries were used to interface with a Quadro P6000 GPU (NVIDIA Corp., Santa Clara, CA). Because the RTT routine requires many distinct transmit and receive events, simulation is computationally expensive. By modeling a linear phased array transducer in 2D, we were able to examine RTT within reasonable computational time limits of about 40 min for a full correction routine.

Two identical transducers were defined, each comprising 96 elements of width 0.245 mm and kerf 0.295 mm, geometries intended to mimic the P4-1 linear phased array transducers (Advanced Technology Laboratories, Bothell, WA) that were used in the experimental section of this study. The transducers were interfaced onto a 315 × 440 (lateral × axial) 2D grid with an isotropic grid point dimension of 0.25 mm (total grid size 78.8 × 110 mm), for a minimum of 6 points per wavelength at 1 MHz and 1500m s^−1^ speed of sound. The kWaveArray class was employed to define the sources, minimizing artifacts caused by placement of partially off-grid sources on a discrete simulation grid (Wise et al., 2019). This layout was chosen to match the ratio of the transducer width and the axial separation between the transducers to that of the geometry in Riis et al. (2024a). The P4-1 transducers were chosen because the ratio of their width to the diameter of the rhesus macaque skulls also approximately matched the ratio of the transducer width to the human skull diameter in the same work. Additionally, the shorter acoustic wavelength at 1–2 MHz was paired with the thinner primate skull, which may help to more accurately probe the case of a lower frequency in a thicker human skull.

It was determined experimentally that the obtained P4-1 transducers had greatly improved sensitivity above 2 MHz driving frequency. Therefore, in the experimental section, the transducers were driven at 2 MHz. However, we ran all parameter-varying simulations at 1 MHz to reduce the necessary grid size and to better match the lower frequencies typically used in tFUS. For the simulation group intended to match experimental parameters, the frequency was accordingly changed to 2 MHz and the grid point dimension was changed to 0.125 mm. The time step was defined in both cases to give a Courant-Friedrichs-Lewy number of 0.3.

**FIG. 1.**
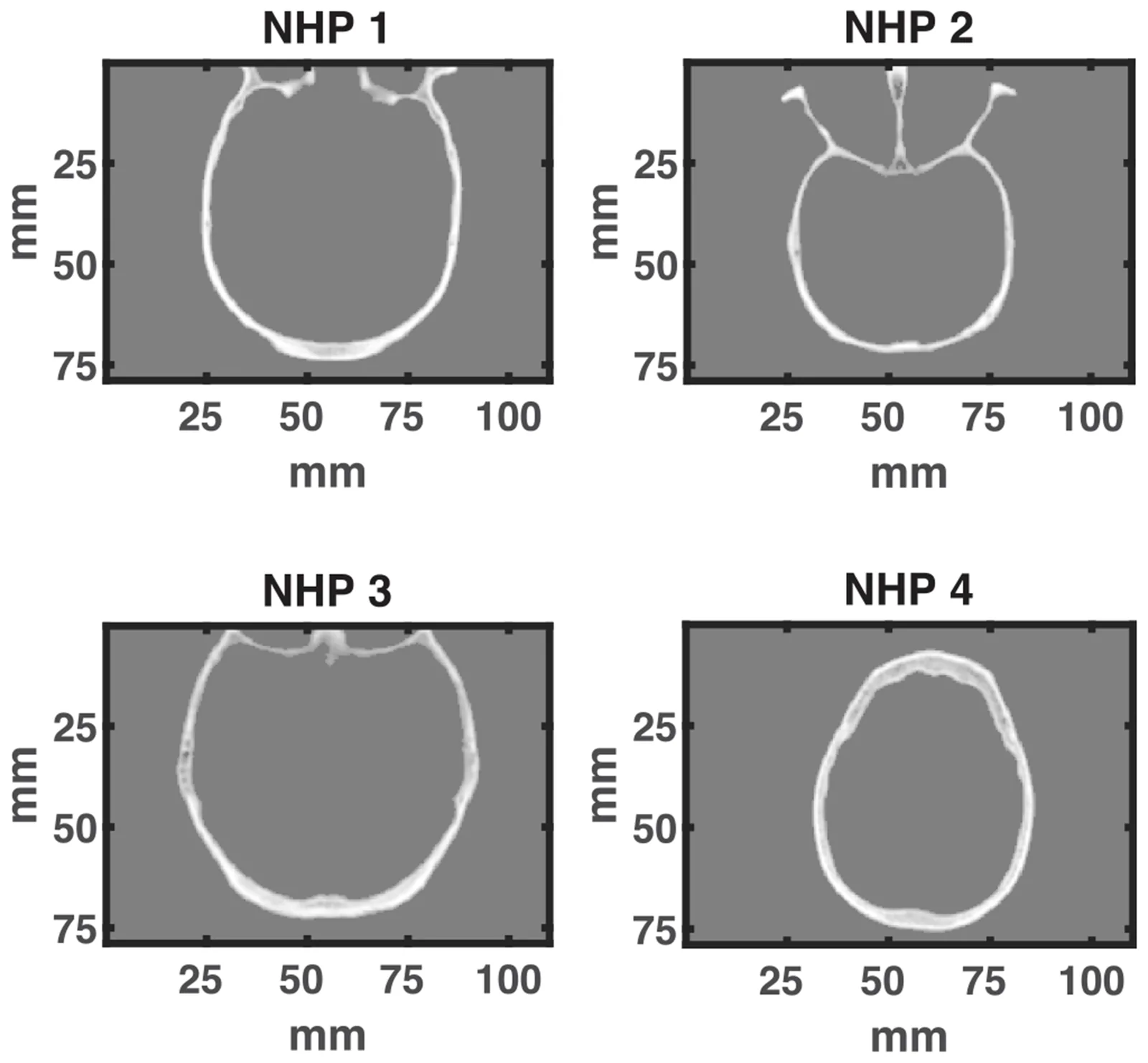
The four transverse slices of CT images of NHP skulls used in all simulations. Brightness is proportional to Hounsfield units and is normalized for each image individually.

To simulate the effectiveness of RTT in use with a live subject, CT images of rhesus macaque monkey skulls that were previously acquired on a clinical scanner (Philips Vereos, Philips Healthcare, Best, The Netherlands) were used to derive the acoustic properties of simulation spaces. A single transverse slice from each image was chosen for intersubject variation in size and shape of the skull. The specific slices used to define the simulation media are shown in Fig. 1. Attenuation α was modeled by power law as $\alpha =af^{y}$, where *a* is the absorption coefficient of a pixel in $dB\;{MHz}^{-1}\;{cm}^{-1},f=1\;MHz$, and *y* = 1:1. Speed of sound *c*, mass density ρ, and absorption coefficient *a* were modeled homogeneously in soft tissue ($\rho_{\text{soft}}=1030\;kg\;m^{-3},c_{\text{soft}}=1500\;m\;s^{-1}$, and $a_{\text{soft}}=0.21\;dB\;{MHz}^{-1}\;{cm}^{-1}$) and water ($\rho_{\text{water}}=1000\;kg\;m^{-3},\;c_{\text{water}}=1500\;m\;s^{-1}$, and $a_{\text{water}}=0.002\;dB\;{MHz}^{-1}\;{cm}^{-1}$). A threshold was used for all images to define a bone mask for pixels with CT Hounsfield units (*HU*)> 367. Sound speed and density were defined heterogeneously in bone by first defining porosity of bone **Φ** as follows:

(6) $$\Phi =1-\frac{HU}{max(HU)},$$

and then mapping parameters linearly with Φ according to the work of Aubry et al. (2003) with $\rho_{\text{max}}=2200\;kg\;m^{-3}$, $c_{\text{max}}=3100\;m\;s^{-1}$, and $a_{\text{max}}=8.1\;dB\;{MHz}^{-1}\;{cm}^{-1}$ as in Verhagen et al. (2019).

When quantifying results of RTT performance, such as peak pressure and focal location, a pseudo-continuous wave comprising approximately 90 cycles was simulated to account for standing wave effects possible during therapeutic pulse trains. A cosine ramp was applied to the first four cycles of the wave to reduce source transients. Spline function interpolation was performed to improve the effective spatial sampling frequency of the grid. To validate the upsampling technique, a simulation was performed at a finer resolution matching the upsampled grid (0.125 mm grid step size at 1 MHz). The root mean square error (RMSE) was 4.01% across the entire grid space, and the focus detected at the same location in both simulations.

### C. Position uncertainty simulations

To model the impact of uncertainty in the positions of the transducers on the efficacy of RTT, three groups of simulations were performed. First, 8 simulations were conducted in which the transducers were simultaneously and oppositely translated axially away from their baseline positions, at 0.25 mm intervals between −2 and 2 mm. Both transducers either approached or receded from the skull simultaneously in these simulations. However, the RTT correction was performed as if the transducers were at their baseline positions. Specifically, the baseline positions of the transducers were used when computing free-field focusing delays and when determining which opposing element pairs were within the acceptance angle. The lateral uncertainty group applied a similar procedure, this time moving the transducers laterally in opposite directions over the same uniform interval, so that as one transducer moved anteriorly, the other moved posteriorly.

Next, 20 simulations were run in which a random uncertainty was assigned individually to each transducer and in each dimension from a normal distribution centered on zero with a standard deviation of 2 mm. Any offset value greater than two standard deviations above or below the mean was compressed to ±4 mm.

### D. Steering simulations

To model the performance of RTT when electronically steering the focus, simulations were performed in which the RTT algorithm was used for correction while steering the beam to 25 locations in a 20 mm × 20 mm grid centered at the initial focus (5 mm step). The simulation space from non-human primate (NHP) 4 was used.

### E. Alternative RTT methodologies tested

RTT was also repeated separately under two modifications to the method for the 4 NHP skulls simulated. In the “Hilbert-RTT” method, a Hilbert envelope was computed for each received waveform during the correction procedure, and the cross correlation was computed between the envelopes of the free-field and aberrated signals, rather than from the signals themselves. In the “Modulo-RTT” method, cross correlation was computed from the untransformed signals, but a modulus operation was performed based on the transducer frequency to convert the time-delay values from the original cross correlation to phase values bounded on the interval [0, 2*π*]. These methods still correct for amplitude in the same way as RTT and should not be confused with the phase-only corrections described in Riis et al. (2024a, 2024b).

### F. Ground truth correction in simulations

To better assess the performance of RTT as compared to other methods, “hydrophone” correction was simulated. To do so, a sensor element representing a hydrophone was first defined at the location of the intended focus in the presence and absence of the skull. Next, each transducer element was pulsed individually, and the signals incident on the sensor element were recorded. Cross-correlation and amplitude comparison were used to determine correction vectors to be applied to the transducer driving signals, just as in RTT.

### G. Experimental methods

To examine whether simulations of RTT could effectively represent real performance, an experiment was designed to match the geometry of simulations. A 2.5D skull phantom was designed as an extrusion of the skull mask from the CT plane of NHP 1 shown in Fig. 1, and surfacepreserving quadric edge decimation was performed on the mesh to reduce print complexity while minimizing disruption to the transmission faces. A bracket was included in the base of the phantom to allow mounting to a custom frame. The homogeneous phantom was 3D-printed using a resin printer (Form 3 & White Resin V4, Formlabs, Somerville, MA) and UV-cured at 60°C for 30 min. To determine the acoustic properties of the resin for use in simulation, a solid block of resin was printed. The block was weighed with an analytical balance and sized by 5 caliper measurements in each dimension to determine the density. Speed of sound through the resin was measured by transmitting a plane wave from the P4-1 in a de-ionized water tank and recording the waveform incident on a fiber-optic hydrophone (FOH) (Precision Acoustics, Dorchester, UK) at a distance of approximately 15 cm. For more on the method of acoustic property characterization, see Li et al. (2022). Data from the hydrophone were collected with a computer-controlled oscilloscope (PicoScope 5000, Pico Technology, St Neots, UK) at a sampling frequency of 125 MHz. The hydrophone was translated laterally using a 3D positioning system to approximately maximize the incident pressure in water alone. The through-resin and water-only waveforms were compared by cross correlation to determine the time delay induced by the presence of the block. The homogeneous absorption coefficient of the phantom was assigned by an optimization routine that varied the parameter in aberrated uncorrected k-Wave simulations until good agreement was seen between experimental and simulated attenuation.

For RTT experiments, two P4-1 linear phased array transducers were driven at 2 MHz by the Vantage 256-channel ultrasound transceiver system (Verasonics, Inc., Kirkland, WA). A custom frame was constructed from aluminum rails, and 3D-printed clamshell mounts were used to rigidly affix the transducers with respect to each other. The translational orientation of all components was aligned by time of flight and physical distance measurements.

Once transducer positioning was complete, the FOH was placed at the intended location of the focus, oriented normal to the ultrasound plane and adjusted in elevation to achieve maximal pressure. The water tank was continuously degassed, and the O_2_ concentration was between 5% and 6% during the experiments as measured by a fiber optic oxygen meter (Microx 4, PreSens, Regensburg, Germany). The transducers were focused electronically by determining time of flight from each element to the hydrophone. Fifty averages were performed of a 10-cycle pulse for each element to improve signal-to-noise ratio (SNR). 2D beam maps were collected in all cases by averaging the instantaneous pressure of 10 waveforms per point at an isotropic pixel size of 0.125 mm.

The free-field RTT scan and beam map were performed before affixing the skull to the custom frame. Once the skull was accurately positioned, the transcranial RTT scan was performed and the uncorrected and corrected foci were beam mapped.

In a second experiment, the beam was also electronically steered to 5 and 10 mm axially and laterally (4 additional locations total) and RTT was performed for each location.

### H. Quantification of results

For simulations, results were quantified by the spatial peak pressure and the focal shift. Spatial peak pressure was defined as the pressure at the grid point where the maximum pressure was achieved after masking and interpolation, and reported pressures were normalized to the simulated free-field spatial peak pressure achieved under geometric focusing. Thus, we define the “intended pressure” as the peak pressure of the free-field focus. Focal shift was defined as the Euclidean distance between the intended focal coordinate and the coordinate of the actual spatial peak pressure. Focal shift analyses were repeated using the unweighted centroid of the −3 dB focal region, and results were comparable in all cases.

## III. RESULTS

### A. RTT performance is variable between subjects

**FIG. 2.**
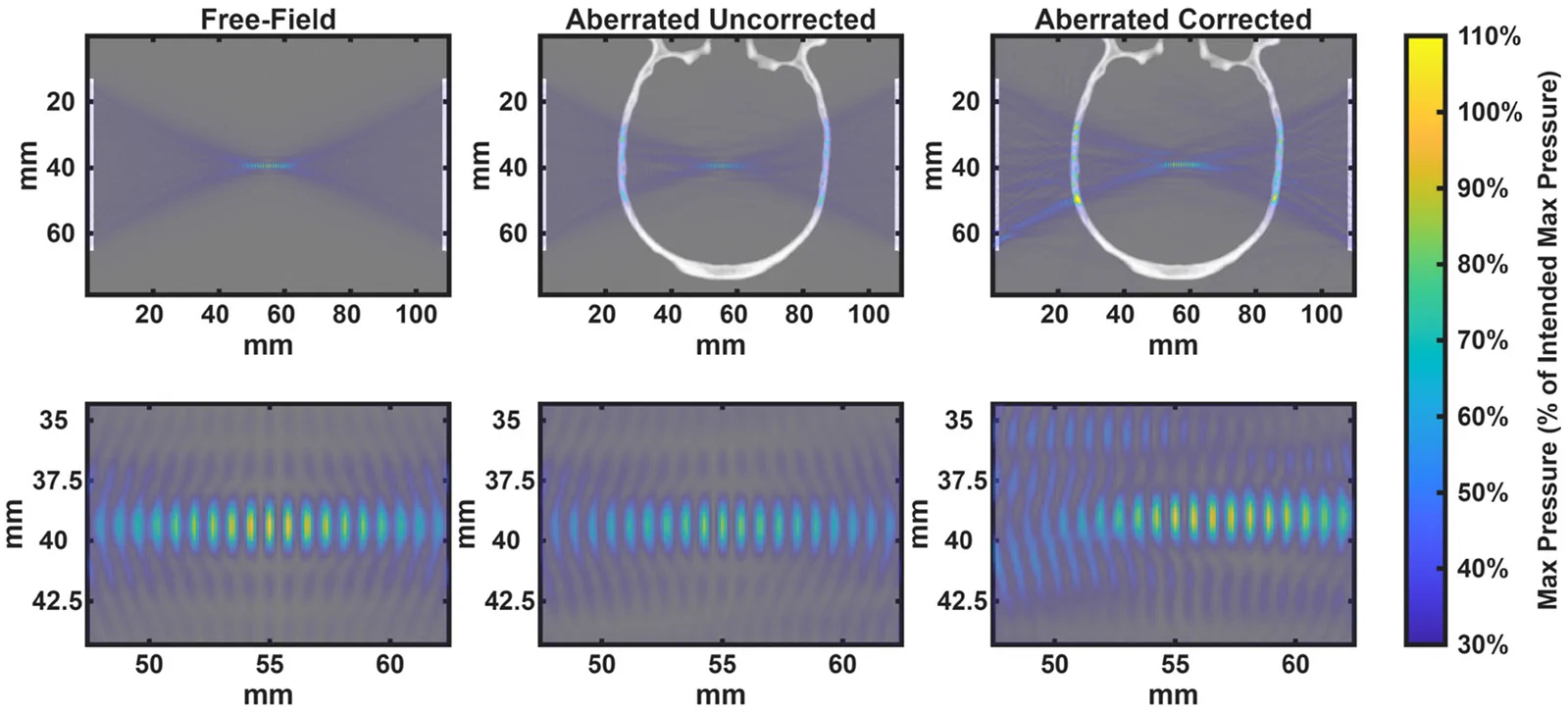
Top: Simulated peak pressure fields in water (left), transcranially without aberration correction (middle), and after RTT correction (right). Bottom: Zoomed focal region of above pressure fields. A standing wave pattern appears due to opposing direction of the transducers, which are depicted in white.

**FIG. 3.**
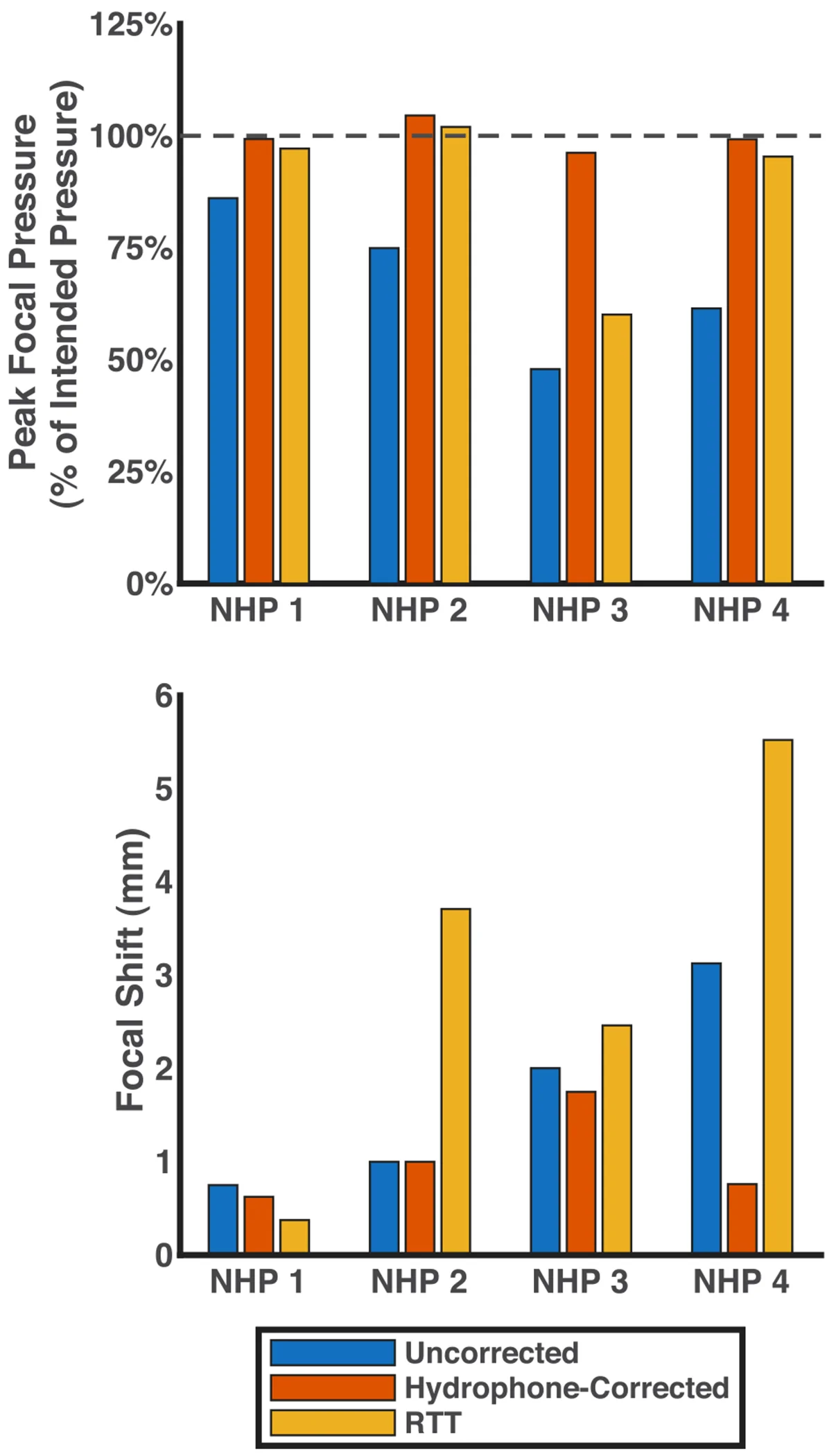
Peak focal pressure (top) and focal shift (bottom) for the 4 NHP skulls simulated without correction, with hydrophone correction, and with RTT correction. Dashed line indicates ideal pressure restoration, and a focal shift of 0 mm means perfect focal location restoration.

RTT was simulated in the four primate skull slices, targeting the center of the simulation grid in each case. An example simulation showing RTT correction is shown in Fig. 2. The free-field −3 dB pressure thresholded focus measured approximately 11.5 mm axially and 1.5 mm laterally at 1 MHz. The skulls were not registered to a specific location within the simulation grid to simulate the impact of head placement for therapeutic targeting of various deep brain locations. The quantification of results for the four skulls is shown in Fig. 3. RTT performance was subjectspecific, delivering within 5% of the intended pressure in three of the four subjects, but only 60% of the intended pressure in NHP 3. Focal shift was also variable and severe across skulls, ranging 0.4 to 5.5 mm. In three of the four subjects, RTT focal shift was greater than the focal shift in the absence of aberration correction. Hydrophone correction, intended to represent the ground truth, averaged 1.0mm focal shift and 100% of the intended peak pressure. Compared to RTT, hydrophone correction achieved a lesser focal shift in three of the four cases and a pressure nearer to 100% of the intended value in three of four cases.

### B. Acceptance angle impacts RTT performance

**FIG. 4.**
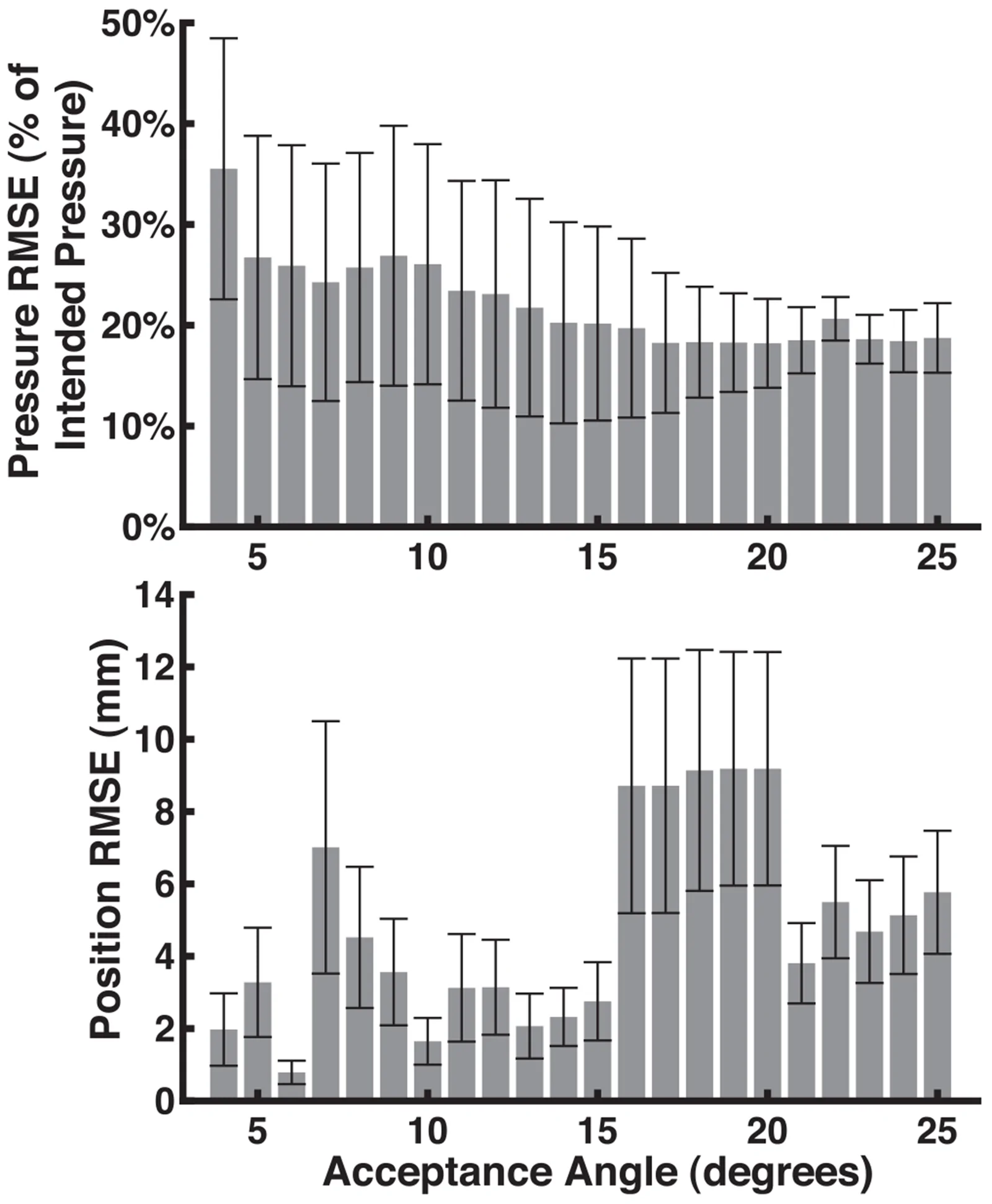
Impact of the acceptance angle on RTT performance in simulation. RMSE for focal pressure (top) and spatial shift (bottom) across all four skull CT images simulated. Bars represent ±1 standard deviation.

RTT was modeled while varying the acceptance angle between 4 and 25 degrees for all four skulls. The RMSE for pressure and focal location are shown in Fig. 4.

For all other simulations and experiments, an acceptance angle of 15 degrees was chosen to provide a balance between focal shift and pressure error.

### C. Position uncertainty weakly impacts RTT performance

The results of position uncertainty simulations are reported in Table I. In the random uncertainty simulations, there was insignificant correlation between the total Euclidean position error and the percent pressure error and focal error (R^2^ = 0.04 and 0.006, respectively).

### D. RTT may fail when steering

**FIG. 5.**
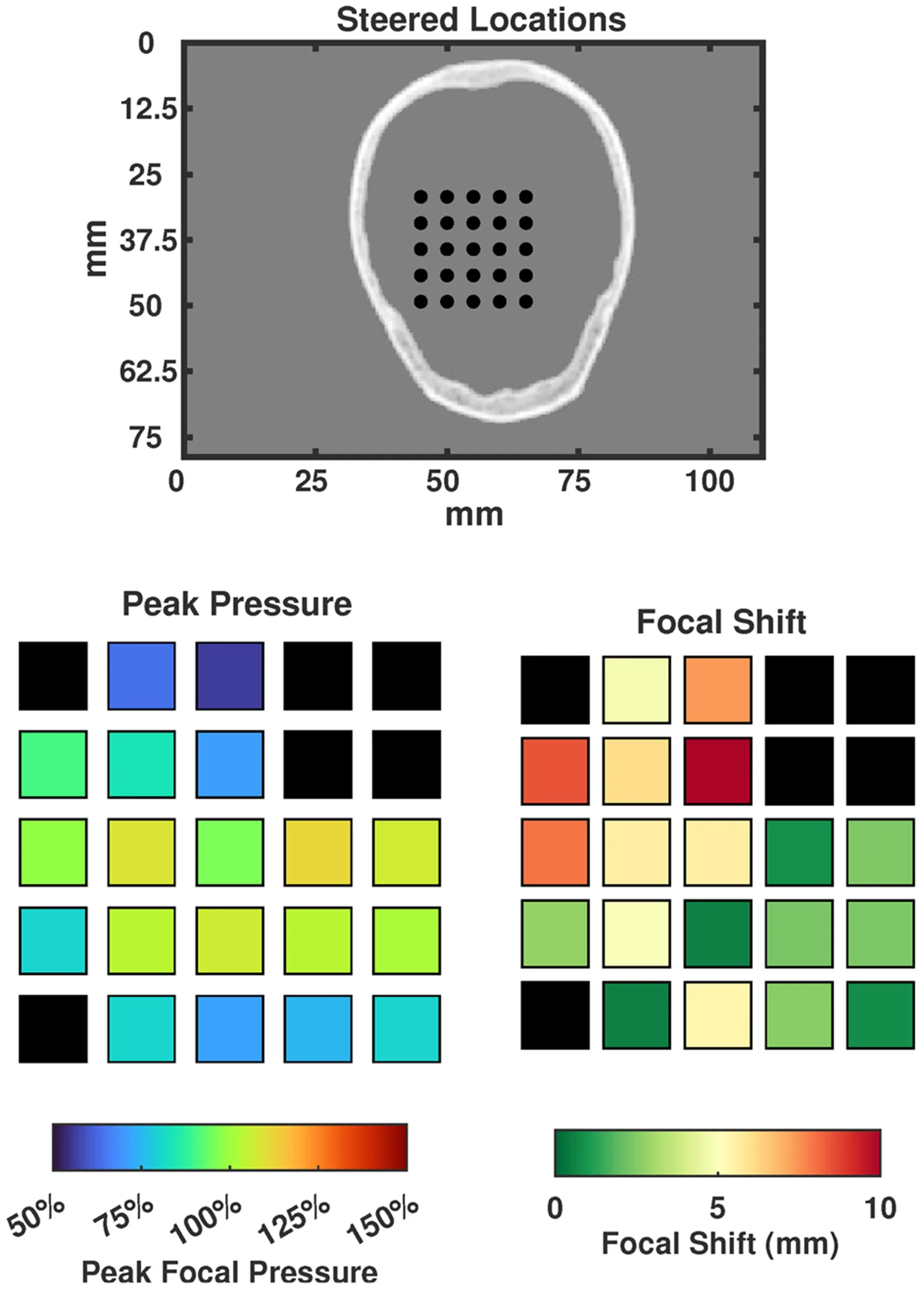
Results of simulating RTT performance when steering to 25 points (black circles, top) in a 20 × 20 mm grid within the NHP skull. Bottom: Peak pressure (left) and focal shift (right) shown in color by grid point. Black represents a failed correction, where the peak pressure occurred in the skull or ≥10 mm away from the intended target.

RTT performance was highly sensitive to steering (Fig. 5). When steering the focus in simulation to 25 locations in a 20 mm × 20 mm grid centered at the initial focus, the location of the focus often differed greatly from the intended location. A failed correction was defined as one where the focal shift was ≥10 mm. Six of the 25 steered coordinates resulted in a failed correction. Of the 19 remaining points, the mean focal shift was 4.2 mm (σ=2.9mm). The mean focal pressure for the locations that did not fail was 89% (σ=17%).

Axial steering had a lesser impact on RTT than lateral steering. For axial-only steering, the mean focal shift and focal pressure were 4.4 mm and 104%, respectively, compared to 5.7 mm mean shift and 80% mean pressure for lateral-only steering.

### E. Low SNR reduces RTT-corrected peak pressure

**FIG. 6.**
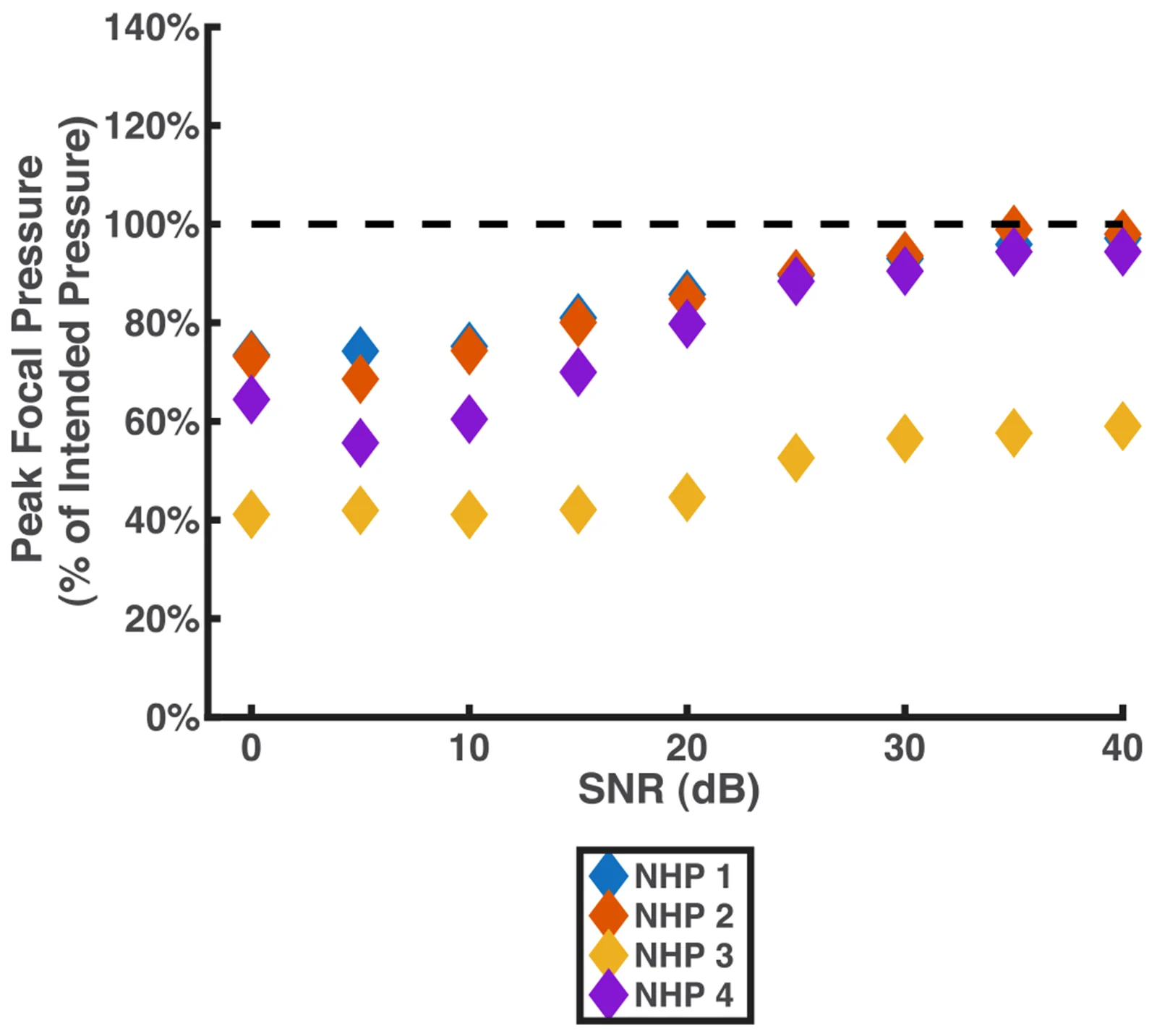
Peak focal pressure achieved after RTT correction under varying SNR in the received signals on each transducer element during the correction process. Dashed line indicates ideal correction.

To characterize the impact of SNR on the effectiveness of RTT, Gaussian white noise was added to each received signal in both the free-field and aberrated cases before the RTT correction was computed. SNR was defined as follows:

(7) $$\text{SNR}=20\;{\text{log}}_{10}\left(\frac{\max\left(s\right)}{\bar{w}}\right),$$

where **s** is the noiseless 10-cycle pulse received on a given element and $\bar{w}$ is the mean amplitude of the added white noise. Simulations were performed with SNR between 0 and 40 dB at 5 dB increments. The results are shown in Fig. 6.

### F. Alternative phase-finding techniques may improve RTT performance

**FIG. 7.**
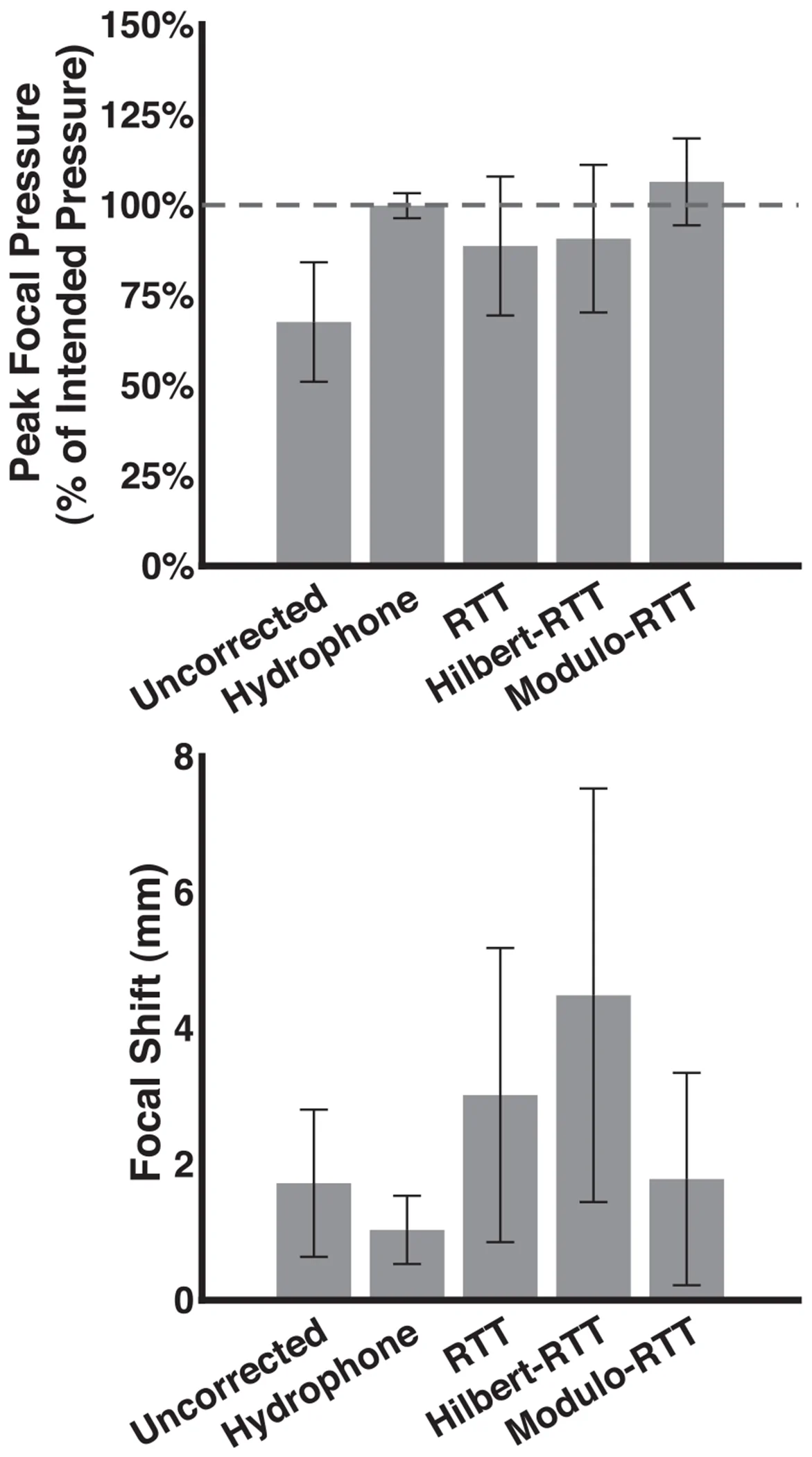
Average peak focal pressure (top) and focal shift (bottom) for the four NHP skulls simulated, by correction method. The 3 RTT conditions are identical in amplitude correction vectors, differing only in the method of computing the phase. Vertical bars show $\pm \sigma /\sqrt{N}$.

The peak pressures and focal shifts resulting from the proposed methodological modifications are plotted in Fig. 7. Paired *t* tests did not reveal significant differences between the hydrophone correction and any of the RTT methods in either peak pressure recovery or focal shift. However, the failure to statistically detect differences may be a result of the low power of this test given the small sample size (*n* = 4), and it is therefore still noteworthy that the mean focal shift was lesser in Modulo-RTT compared to RTT and Hilbert-RTT. Additionally, the variances in peak pressure for both RTT and Hilbert-RTT, but not Modulo-RTT, were significantly greater as compared to hydrophone correction by an *F* test (p < 0.05).

Since the three RTT methods differ only in computation of the phases τ (i.e., the correction amplitudes **x** are the same across all three), it is worth mentioning that RTT returned an average element-wise amplitude correction factor that was 17.4% greater compared to the hydrophone correction. Despite this, the mean peak pressure delivered transcranially was, compared to hydrophone correction, 11% lower in standard RTT, 9% lower in Hilbert-RTT, and 6% higher in Modulo-RTT. Thus, while none of the RTT methods was as efficient as hydrophone correction in terms of transcranial pressure delivered per average element amplitude, Modulo-RTT computed phases that improved focal gain compared to the naive approach.

### G. Experimental results match pattern of simulations

The measured acoustic parameters of the 3D-printed phantom are given in Table II. The simulated absorption coefficient α was optimized such that the pressure transmission through the skull in simulation matched the experimental result to a difference of <1%. This was done to reduce the contribution of error in parameter estimation to the differences between simulation and experiment.

**FIG. 8.**
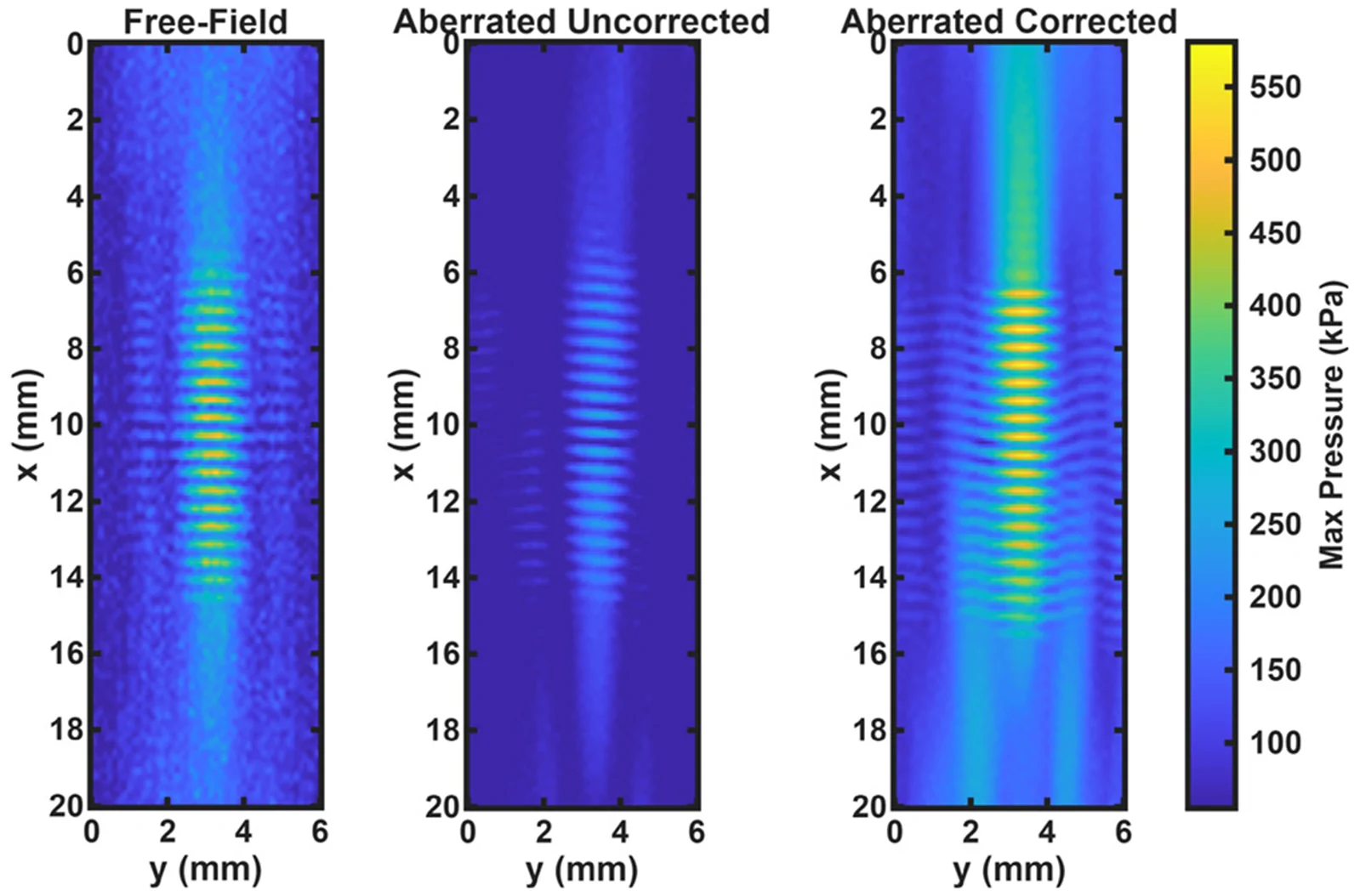
2D beam maps of recorded peak negative pressure in water (left), through the skull phantom without aberration correction (middle), and after RTT correction (right).

Beam maps from the experimental implementation of RTT are shown in Fig. 8. In free field, the −3 dB pressure thresholded focus measured approximately 6.4 mm axially and 1.2 mm laterally.

In both simulation and experiment, the transmitted focal pressure without correction was 63.6% of the free-field pressure. The uncorrected focal shift was 0.2 mm in experiment and 0.4 mm in simulation. After RTT correction, the focal pressure in experiment was restored to 122.4% of the free-field pressure with a focal shift of 0.3 mm. 2D beam maps of the three foci are shown in Fig. 8. In simulation, RTT correction restored 91.9% of the intended pressure with a focal shift of 0.4 mm.

The peak pressures achieved when steering the beam in the experimental implementation of RTT were similar to those achieved in the simulated implementation. When steering laterally to 5 and 10 mm, RTT restored 96% and 78% of the intended pressure, respectively. When steering axially at the same intervals, RTT restored 94% and 110% of the intended pressure. In the experiment and the simulation designed to match it, the magnitudes of focal shifts during steering were much lesser than in the simulations at 1 MHz with heterogeneous digital phantoms. The experimental shifts averaged 0.8 mm when steering laterally and 0.5 mm when steering axially.

## IV. DISCUSSION

### A. Acceptance angle and steering

The apparent trend in the geometry simulated was a reduction in the RMSE of peak pressure with increasing acceptance angle until approximately 16°, above which the pressure correction error remains almost unchanged. However, the RMSE in focal position increased at acceptance angles greater than 15°. Since the reduction in peak pressure error was only modest after 15°, this value was chosen for the remaining simulations to balance between ideal focal shift and focal peak pressure and to maintain consistency.

Contrary to the results reported in Riis et al. (2024b), this work demonstrates that the RMSE for both focal shift and peak pressure is sensitive to acceptance angle. While this result may be explained by the difference in transducer geometry in our study, it implies that, for any implementation of RTT, thorough testing and optimization of the acceptance angle should be performed before use. We emphasize, however, that we do not recommend the use of this particular acceptance angle as a general-purpose value for RTT. Rather, any given implementation of RTT should be tested to determine what acceptance angle provides the best performance with respect to focal location and pressure accuracy.

Targeting specific brain structures with millimeter precision is highly desirable for tFUS therapy and research. This necessitates a high degree of control and accuracy in electronically steering the focus. The noteworthy variability in performance (and sometimes complete failure) of RTT under steering therefore represents a challenge for the method.

Our results, both experimental and simulated, show that on-axial steering has a lesser impact on RTT performance than does off-axial steering. The experimental and simulated results both show pressure restoration errors >10% when steering, although the results differ in the magnitude of the focal shift observed. This may be due to the difference in speeds of sound and densities of simulated bone and the 3D-printed phantoms.

Of note, the results we report concerning the impact of steering and acceptance angle are likely to be more sensitive to the geometry of the transducers than other results (e.g., the impact of SNR), and warrant further study in large therapeutic array transducers, especially hemispherical transducers where transmission efficiency is improved due to the directivity of individual elements coinciding with their angles of incidence on the skull.

Considering these results, we suggest that future work should examine the impact of acceptance angle in RTT on the ability of the method to accurately correct in steering.

### B. Position uncertainty and noise

For a single-transducer tFUS setup, a given uncertainty in the position of the transducer will translate to some uncertainty in the position of the focus and possibly in aberration correction methods that rely on knowledge of element position, such as ray-tracing. However, in the dual-transducer setup, a position error of *λ*/2 will cause destructive interference in the region of the standing wave. Therefore, registration between the two transducers is of great importance in any dual-transducer setup in order to maintain the intended focal size, pressure, and location. At high frequencies, this problem requires maintaining submillimeter precision, or correcting for position error. The results presented in this work show that RTT itself appears to be robust to small random uncertainties in transducer positions, as demonstrated by the 91 ± 8% pressure delivery on average for the 20 random-uncertainty simulations. As with steering, on-axial position uncertainty has a lesser impact on RTT performance than off-axial uncertainty. This result may be explained by the linear geometry of our transducer arrays, because a given shift in the axial dimension will translate to a lesser misalignment in opposing transducer elements than an equivalent lateral shift. Regardless, the simulated and experimental results in this work add support to the conclusion that accurate transducer positioning is important in dual-transducer setups not just for targeting, but also for delivery of pressure.

Another implication is that lower transmit frequencies will be more robust to positioning error in dual-transducer methods (including RTT), since *λ*/2 will be increased and thus the proportionality constant of the peak pressure to the position uncertainty will be reduced.

Of note with respect to the SNR of the received signals in RTT is that the focal pressure is nearly always reduced by the presence of modest noise. While a reduction in focal pressure is bad in the sense that it means the correction is imperfect, for safety purposes, it is noteworthy that large increases in peak pressure do not seem to occur as a result of signal noise. For relatively high SNR (>20 dB), this pressure reduction appears to occur primarily due to the observed tendency of the RTT-computed phases to approach a uniform random distribution as noise is added to the input signals. Below an SNR of 10 dB, where the magnitude of white noise can begin to exceed the maximum amplitude of the pulse peak, the RTT-computed correction amplitudes will also tend to approach a mean of 1 (i.e., no amplitude increase), since in the limiting case the maximal amplitudes of the free-field and transcranial signals will be determined by the (equal) noise in the signals. In future work, digital bandpass filtering may help to reduce the apparent high-frequency noise in the signals, but care should be taken to avoid introducing phase shifts in the signals which could adversely impact the quality of the correction.

### C. Agreement between experiment and simulation

The experimental results show that the physical implementation of RTT yields aberration correction performance within the range of simulated performance. This demonstrates that RTT can be implemented in 2D and in the geometry simulated, thereby validating a primary assumption made in simulations. However, we do not see, and should not expect, perfect agreement between simulation and experiment. Although care was taken to maintain alignment between simulation and experiment, the sub-millimeter precision required to perform a true one-to-one comparison between simulation and experiment was not likely achieved. Moreover, our simulations do not consider nonlinear propagation and shear waves, and are subject to other uncertainties that arise in the many simplifications necessary for simulation. Finally, experimental uncertainties, such as the manufacturer-reported ±8% uncertainty on the FOH calibration, could not be mitigated through repeated trials and may also contribute to the discrepancy between experimental and simulated results.

In the experimental implementation of RTT and the simulation of the resin phantom, the average magnitude of focal shifts was much smaller than in the simulations. We propose that this discrepancy is a result of the different material properties of the 3D-printed skull phantom as compared to those of a real skull. Based on the measured speed of sound and density of the phantom, the characteristic acoustic impedance of the skull phantom is approximately half that of the maximum impedance in the CT-derived simulated skull (3.0 × 10^6^ Rayl for the phantom, 6.8 × 10^6^ Rayl for the CT-derived simulation medium). Perhaps more importantly, the skull phantom is highly homogeneous internally due to the use of a resin printer (as opposed to an extrusion printer) in its fabrication. This is distinct from the interior of a real skull, whose microstructural heterogeneity has been shown to play a significant role in acoustic propagation (Pinton et al., 2012; Robertson et al., 2018). While our CT images do not capture the microscopic detail of the trabecular bone, they do display at least some degree of internal heterogeneity, in contrast to the homogeneous phantom. Finally, the simulations do not account for acoustic lensing material on the transducer that may improve their tendency to form a natural focus.

Last, although our simulation methods are in line with established state-of-field guidelines (Aubry et al., 2022), our simulations do not perfectly model every aspect of acoustic propagation through a skull. One important limitation of our work is that our fluid simulation model does not account for shear wave mode conversion as a viscoelastic model would. Our optimization of acceptance angle may be impacted by shear wave propagation as the angle of incidence increases. Viscoelastic modeling has been shown to affect the accuracy of focal pressure and volume estimates in tFUS simulations (Mueller et al., 2017). Future numerical work should investigate the impact of shear wave propagation on RTT. Staircasing at material boundaries can also introduce reflections and other numerical errors to simulations (Drainville and Pichardo, 2020), and future studies should consider implementing advanced techniques to avoid these and other uncertainties. Nevertheless, simulation remains the best tool to study individual aspects of RTT or any correction method, since parameters can be controlled exactly. Moreover, simulation methods similar to those employed in our work have been thoroughly studied and shown to provide results that are useful in studying reality with a tolerable degree of error (Krokhmal et al., 2025). Thus, since our experimental results showed good agreement with the simulation of the homogeneous phantom, and since our simulations of heterogeneous phantoms are in line with the state of the field, we believe our experiment adds justification to the simulation results of this study.

### D. Impact of phase-finding technique

Intuitively, it might be expected that a time-delay, rather than a true phase, would be the required input to the pseudoinverse (as $\tau_{i,j}$). This is because the physical meaning of the system of equations set up in Eq. (3) (when solving for τ) is that the total speedup experienced by any given wave as it passes through the skull is a sum of the speedup component experienced on each side of the skull individually. Thus, it is natural that the pseudoinverse should separate these values with units of time, rather than units of phase.

However, using time in the pseudoinverse rather than phase results in poorer performance, as shown in Fig. 7 (“RTT” vs “Modulo-RTT”). This is likely caused by cycle skipping in the “RTT” group. If, for example, $\tau_{i,j}$ was computed to be exactly one cycle longer (i.e., if the cross correlation had skipped one period) as compared to $\tau_{i,j+1}$, then the contributions from each of these values in the pseudoinverse for $\tau_{i}$ would average to an intermediate value that is exactly out of phase with the ideal value. However, if all the $\tau_{i,j}$ are treated as phases, bounded by a modulus between 0 and 2*π*, this issue is avoided and the results improve accordingly.

In Fig. 7, it is important to note the amplitude vector **x** for the three RTT conditions is identical; only the phase vector τ is computed differently. The results therefore indicate that the phases found by the “Modulo-RTT” method provide the most efficient superposition of pressure at the focus and also lead to the least average focal shift of the noninvasive methods.

Short (e.g., half-cycle) pulses could provide a sharper cross correlation function and thus allow for improvement of results without computing a phase; however, therapeutic transducers, especially at low frequency, may show significant ringing before a steady-state is achieved, thus distorting the results of RTT. Further work should investigate whether varying the number of cycles in RTT, perhaps using a different number for the amplitude and phase corrections, could improve RTT performance.

### E. Generalization to three dimensions

Simulations of RTT were limited in this work to a 2D implementation in order to simplify the system for the analysis performed. In experiments, the setup was 2.5-dimensional in the sense that, while it occurred in real 3D space, the linear phased array transducers and extended skull phantom mimicked a 2D setup. However, we expect that the methodological improvements demonstrated would generalize to the 3D case. Because the transducer element inputs to the pseudoinverse method are entirely vectorized, the dimensionality of the problem does not affect the computation. We believe that this 2D study also provides evidence about general trends in RTT performance that may affect the 3D array case. However, there are likely differences in the 3D case that warrant further investigation; the observations presented in this paper should guide future exploration into the full implementation of RTT. While an in-depth simulation-based study of RTT in 3D would incur prohibitive computational cost with the methods employed herein, frequency-reduction methods, such as those described in Maimbourg et al. (2020), could be used to significantly improve computational efficiency.

### F. Transmission efficiency in RTT and other aberration correction methods

The results showed that RTT may not achieve transmission efficiency that is comparable to that of the hydrophone, in the sense that more energy is transmitted for the same pressure achieved at the focus. This implies that more absorption and undesirable heating may be occurring in the skull when RTT is applied. However, it is also worthwhile to remark that RTT and hydrophone methods in general are not concerned with the efficiency of transmission, but rather with knowledge of the peak pressure attained at the focus. Precise knowledge of the pressure at the focus is important for therapeutic and safety considerations (Aubry et al., 2025), but highly lossy transmission may lead to significant skull heating. Assuming the skull is heterogeneously attenuating, the hydrophone and RTT methods will both increase the driving amplitudes for elements transmitting through the most highly attenuating sectors of the skull, resulting in higher energy absorption in these same sectors. The same would also apply to typical ray-tracing methods.

In future work, an improved aberration correction method could, rather than focusing on individual element-wise amplitude adjustments, take a global approach to the problem. By considering the total energy that is attenuated by the skull across all elements, a global method might improve transmission efficiency.

### G. Subject-specific performance

The results show appreciable variability in RTT performance for different subject skull geometries (Figs. 3 and 7). No obvious relationship was observed between the correction quality and the average angle of incidence of ultrasound on each skull, suggesting that internal skull heterogeneity or other factors contribute to the variable performance of RTT across subjects. The range of pressures delivered is also of interest (Fig. 3) because an overdelivery of pressure represents a safety concern and an underdelivery may lead to a failed treatment. Notably, much of the improvement seen in Hilbert-RTT and Modulo-RTT came from an increase in pressure delivery in NHP 3, for which the standard RTT method had only delivered 60% of the intended pressure. In this case, Hilbert- and Modulo-RTT restored 60% and 94% of the intended pressure, respectively. However, these methods of computing the phase correction also led to more efficient transmission in other NHPs, such that the delivered pressure was higher than intended in some cases (up to a maximum of 119% of the intended pressure in NHP 2).

Methods should be explored in future work to non-invasively determine how effective an RTT correction has been for a particular subject. Without employing imaging-based methods (such as magnetic resonance acoustic radiation force imaging) or simulation-based methods, it may be difficult to estimate the efficacy of a particular RTT correction even *a posteriori*. Riis et al. (2024a, 2024b) used the total coherence of corrected pulses with their free-field equivalents as a metric for phase correction quality. Future work could explore different metrics to estimate quality in light of some of the parameter sensitivities reported in this work.

### H. Safety in RTT

Ensuring safety in tFUS requires consideration of thermal and mechanical bioeffects of ultrasound (Aubry et al., 2025). Mechanical bioeffects are most relevant at the focus, while thermal effects also involve the skull and scalp. Our results are consistent with Riis et al. (2024a, 2024b), indicating that RTT may occasionally overcompensate for aberration and deliver peak pressure that is greater than intended, in some cases exceeding the intended value by more than 15%. In light of this, the RTT method should not be used without stringent, conservative upper limits on pressure delivery. We advocate for a derating scheme that is robust to steering, uncertainties, noise, and anatomical variability. Because RTT increases the individual element amplitudes, it may be important to ensure that no element be driven at an excessive intensity. The specifics will necessarily be system-dependent, and should be based on accepted standards for estimating temperature and pressure or statistical estimation of population upper-limit transmission (e.g., Aubry et al., 2025; Attali et al., 2023).

## V. CONCLUSION

An existing aberration correction method (RTT) for tFUS that does not rely on prior imaging to compute phase and amplitude corrections was characterized in simulation and experiment. Simulation could not perfectly replicate experiment, but RTT did perform within the expected range in both simulation and experiment, lending justification to the *in silico* approach. RTT regularly restored the intended pressure at the focus but did not always restore the focal location. Computing phase corrections instead of time delay corrections as inputs to the matrix equations seemed to improve the correction method, and especially reduced the intersubject variability of performance. RTT was sensitive to the acceptance angle criterion and to steering, but was more robust to physical uncertainties. Noise in received signals generally reduced the focal pressure achieved after correction. Future work should investigate the impact of the acceptance angle and phase finding method on the steering performance of RTT, as this was an area of high and unpredictable sensitivity.

## Supplementary Material

S1-S4

See supplementary material for Figs. S1–S4, which give additional details about numerical inputs and outputs of RTT, such as the impact of Tikhonov regularization and singular value truncation.

## Figures and Tables

**TABLE I.** Results of 20 simulations where position error was applied to both transducers up to 4 mm in each dimension before performing RTT.

	Focal pressure (% of intended)	Focal shift (mm)
Axial uncertainty		
Mean	96	4.9
σ	3	0.6
Minimum	89	4.0
Maximum	99	5.5
Lateral uncertainty		
Mean	87	6.8
σ	7	4.9
Minimum	81	1.6
Maximum	98	14.5
Random uncertainty		
Mean	91	4.7
σ	8	3.5
Minimum	76	0.5
Maximum	107	11.7

**TABLE II.** Experimentally determined speed of sound, density, and absorption coefficient of the resin phantom.

*c*_resin_ (ms^−1^)	ρ (kg/m^−3^)	α (dB MHz^−1^ cm^−1^)
2577	1167	2.54

## Data Availability

The data that support the findings of this study are available from the corresponding author upon reasonable request.
