# Supplementary material for: Performance of a through-transmit aberration correction method for transcranial focused ultrasound: Simulation and experimental evaluation
Source: J Acoust Soc Am. Author manuscript; Available in PMC 2026 Aug 27. (PMC13520834; doi:10.1121/10.0043031)
Supplement: Supplementary file 1 [file NIHMS2199434-supplement-S1-S4.zip › SuppPub1.pdf]

Supplementary Material for "Performance of a Through-Transmit Aberration Correction Method for Transcranial Focused Ultrasound: Simulation and Experimental Evaluation"

Cooper L. Donovan and Charles F. Caskey, Ph.D.

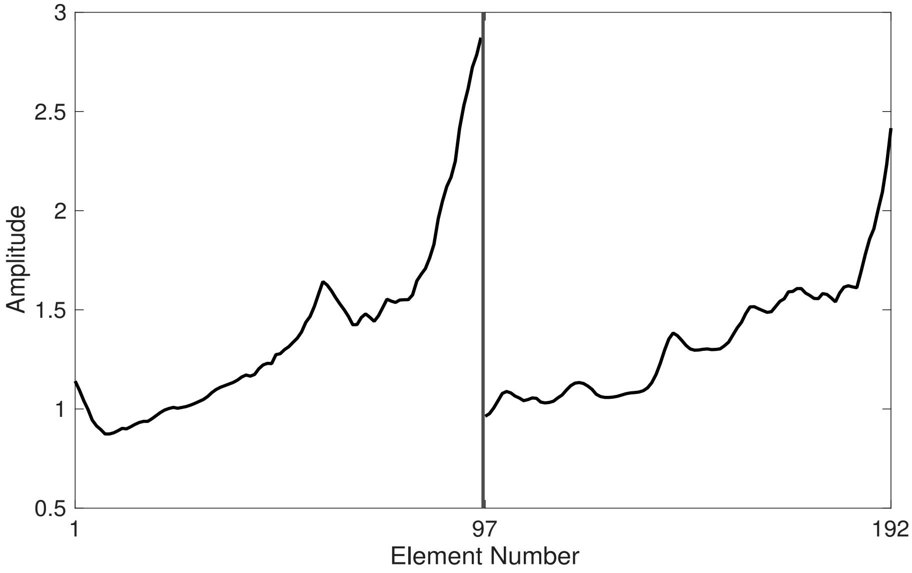

Figure 1: Example amplitude correction vector computed by RTT for Subject 1. Elements 1-96 are of the first transducer and elements 97-192 are of the second. An amplitude of 1 represents the same driving amplitude as in free field.

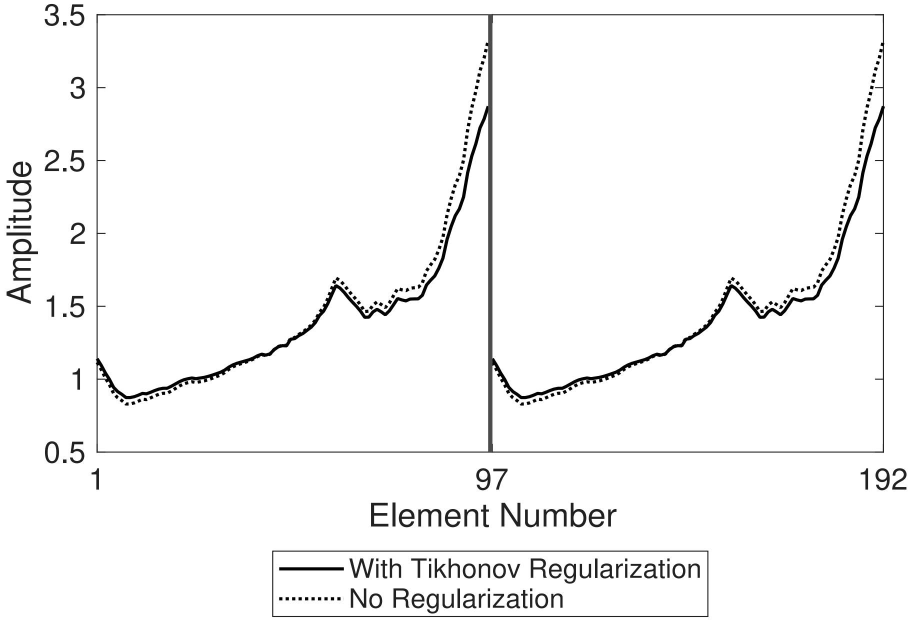

Figure 2: Same as Figure 1, but with dotted line displaying the result of performing the pseudoinverse without Tikhonov regularization. Note the smoothing effect of regularization, where small amplitudes are amplified and large amplitudes are muted. The parameter λ is chosen such that the generalized cross-validation (GCV) is maximized.

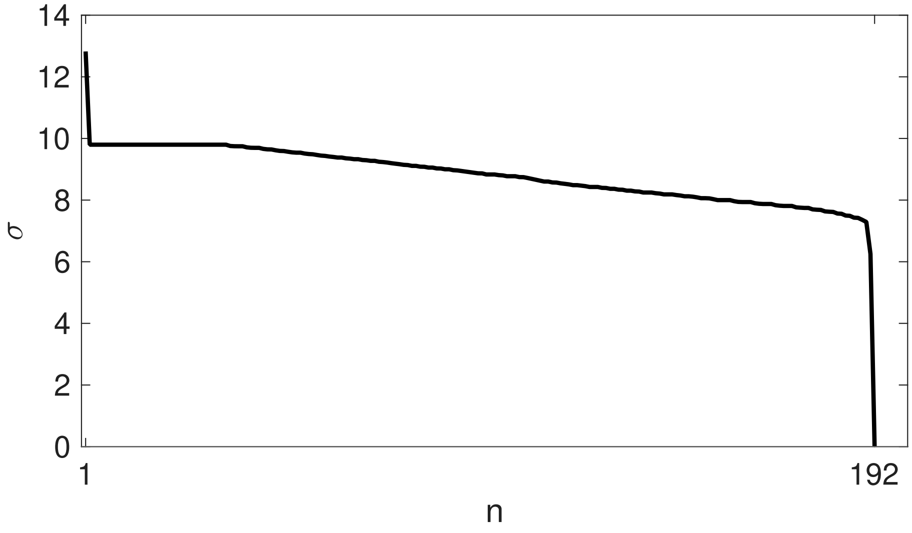

Figure 3: Sample singular values for the pseudoinverse of one half of the weighted A matrix at a 15-degree acceptance angle. For $n = 192$, $\sigma \approx 10^{-15}$, which was less than the truncation threshold $m^2 \times \epsilon \times \|A\| \approx 10^{-11}$, where $m = 96$ and ϵ was the machine tolerance.

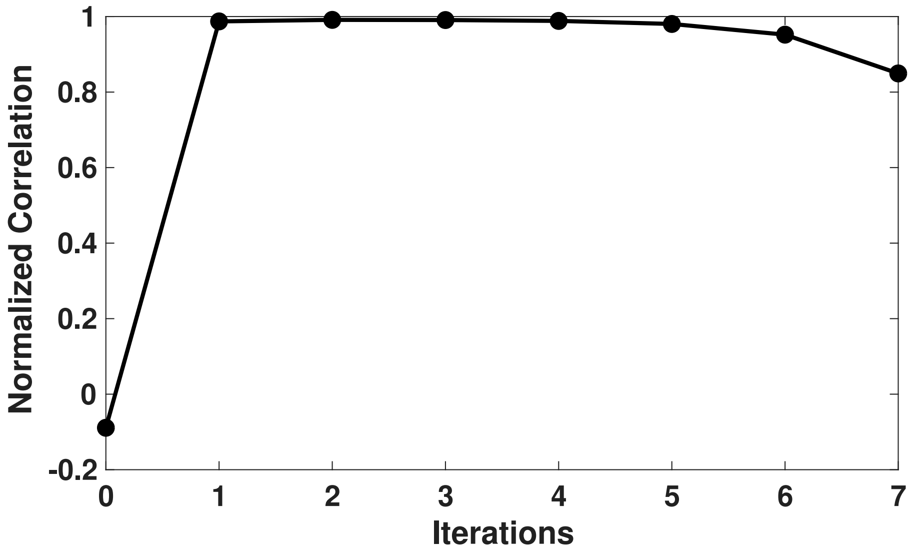

Figure 4: Plot demonstrating instability in the iterative RTT method for a single subject (NHP 4). The normalized correlation C across all elements is plotted as a function of the iteration number, where iteration 0 is the initial, uncorrected correlation. In this case 98.7% correlation is reached in the first iteration. Since no appreciable benefit could be demonstrated when using the phases from iterations with slightly higher correlation, RTT was performed in this work with only one iteration.
